# Matrix Solid-Phase Dispersion Procedure for Determination of Antibiotics and Metabolites in Mussels: Application in Exposure Bioassays

**DOI:** 10.3390/molecules29225478

**Published:** 2024-11-20

**Authors:** Carmen Mejías, Tainá G. Fonseca, Noelia García-Criado, Julia Martín, Juan Luis Santos, Irene Aparicio, Esteban Alonso

**Affiliations:** 1 Departamento de Química Analítica, Escuela Politécnica Superior, Universidad de Sevilla, C/Virgen de África, 7, E-41011 Seville, Spain; cmpadilla@us.es (C.M.); ngarcia5@us.es (N.G.-C.); jbueno@us.es (J.M.); jlsantos@us.es (J.L.S.); iaparicio@us.es (I.A.); 2 Centre for Marine and Environmental Research—CIMA/ARNET—Infrastructure Network in Aquatic Research, University of Algarve, Campus de Gambelas, 8000-139 Faro, Portugal; tgfonseca@ualg.pt

**Keywords:** mussels, antibiotics, metabolites, MSPD, LC-MS/MS, pharmaceuticals

## Abstract

The presence of antibiotics in seafood for human consumption may pose a risk for consumers. Furthermore, some marine organisms, such as mussels, can result in appropriate bioindicators of marine contamination. In this work, a multiresidue analytical methodology suitable for the determination of antibiotics and metabolites in mussels is proposed. The target compounds include three sulphonamides and trimethoprim (TMP) and six of their main metabolites. Sample treatment involves extraction and clean-up in a single step using matrix solid-phase dispersion with acetonitrile. Analytical determination was carried out by liquid chromatography–tandem mass spectrometry. Good linearity (R^2^ > 0.99), accuracy (from 80.8 to 118%), and limits of quantification (lower than 5 ng g^−1^ (dry matter, dm)) were obtained for all selected compounds. The method was applied to the determination of antibiotics in mussel samples from an exposure assay with contaminated seawater with TMP and sulfamethoxazole (SMX). Both antibiotics were detected in the analysed samples with concentrations up to 77.5 ng g^−1^ dm. TMP was bioconcentrated to a higher extent than SMX, attributable to its higher hydrophobicity. None of the metabolites were detected. These results demonstrate that *Mytilus galloprovincialis* is a suitable bioindicator to assess marine pollution.

## 1. Introduction

In 2021, antibiotic consumption in Europe was measured at 125 and 92.6 mg per kg of biomass for humans and food-producing animals, respectively [1]. From 2014 to 2021, antibiotic use in animals raised for food dropped by 44%, whereas, in humans, it stayed relatively constant [1] despite the efforts carried out to reduce the consumption of antibiotics as growing concerns are being raised about their overuse and misuse. After consumption, antibiotics are released into the environment primarily through wastewater treatment plants, including domestic effluents and effluents from industries and hospitals, due to their incomplete degradation. The world oceans are regarded as the final repository for sewage and other human by-products [2]. The most significant antibiotic in the marine environment in terms of its detection frequency and concentration levels is sulfamethoxazole (SMX) [3]. It was detected at various locations, with concentrations of up to 6.3 ng L^−1^ in the Saronic Gulf and the Eleusis Bay [4] and up to 70.1 ng L^−1^ in the Baltic Sea [5]. Trimethoprim (TMP), commonly administered jointly with SMX, was reported at lower concentration levels (concentrations up to 21.8 ng L^−1^ in the Hong Kong Bay [6]) than SMX but with remarkably high detection frequency [3].

The continuous exposure of aquatic ecosystems to antibiotics is an issue of environmental and human health concern, as antibiotics are conceived to exert specific biological effects, even at low concentrations. The increasing environmental occurrence of antibiotics may promote the proliferation and dispersion of antibiotic-resistant bacteria strains that threaten the treatment of infectious diseases [7]. An extensive list of antibiotics has already been found in the tissues of marine organisms, revealing their bioaccumulative potential [8,9]. Residues of TMP and SMX were quantified in mussel tissues at concentrations of up to 9.22 and 13.9 ng g^−1^ dry matter (dm), respectively [8,9]. Therefore, long-term exposure to antibiotics and their transference to higher trophic levels may pose potential risks to marine biota, besides posing negative consequences on human health through the consumption of chemically contaminated seafood [10]. The main public health concern associated with this practice is the human acquisition of antimicrobial-resistant bacteria or genes through consuming contaminated food products, which has been demonstrated previously in mussels [11].

Marine mussels are suitable sentinel organisms to assess the impacts of anthropogenic stressors in coastal waters [12]. They are sessile filter-feeding organisms that can efficiently accumulate chemical pollutants from the surrounding water, providing an integrative measure of the concentration and bioavailability of seawater pollutants [13,14]. Moreover, mussels are globally distributed, easily accessible, and have a high tolerance to a wide range of environmental parameters, including temperature, oxygen levels, salinity, and food availability.

Antibiotics and their metabolites have been extracted from marine bivalves using the QuEChERS method [9], pressurised liquid extraction [15,16], ultrasound-assisted extraction [17], and microwave-assisted extraction [18]. Extract clean-up has been performed by solid-phase extraction [15,16] and liquid–liquid extraction [17]. The determination has been made mainly by liquid chromatography–tandem mass spectrometry (LC-MS/MS) [9]. However, the preparation of samples can sometimes be complex and expensive, leading to time-consuming procedures that require large volumes of solvent and result in poor reproducibility. Therefore, there is a growing need to develop new, reliable analytical methods for determining antibiotics in mussels. Matrix solid-phase dispersion (MSPD) is a technique that integrates extraction and purification into a single step, facilitating the analysis of contaminants in environmental, food, and other complex matrices. The soft tissues of marine animals are indeed complex matrices, containing various compounds like lipids and proteins, which can interfere with detection and quantification processes, impacting both selectivity and sensitivity. In this technique, the solid sample is mixed with a dispersant to achieve complete sample disruption and isolate the analytes. The analytes are then eluted using an appropriate solvent [19]. This technique reduces solvent consumption, extraction time, and sample amount, making the extraction method highly suitable, fast, economical, and simple in terms of sample preparation. MSPD has been applied with success for the determination of bisphenols [20,21,22], phthalates [22,23], pesticides [24], parabens [25], polychlorinated biphenyls [26], herbicides [27], UV filters [28], perfluorinated compounds [29], and flame retardants [30,31] in mussels, demonstrating the suitability of the technique to extract chemical compounds from such biological matrix.

The aim of this work was to develop and validate a new method for the determination of TMP, sulphonamides, and their main metabolites in marine mussels, *Mytilus galloprovincialis*, using MSPD as an extraction technique. This method offers several advantages over other conventional extraction techniques, including reduced time consumption, lower costs, and decreased solvent usage. To the best of our knowledge, this is the first methodology of this approach being applied for antibiotic determination in mussel samples. Furthermore, the methodology was applied to whole soft tissues of *M. galloprovincialis,* exposed to environmentally relevant concentrations of SMX and TMP, in an in vivo 28-day bioassay.

## 2. Results and Discussion

### 2.1. Method Optimisation

The key parameters influencing MSPD, namely the type of extraction solvent, the type and amount of clean-up sorbents, and the number of extraction cycles, were assessed. Optimisation was conducted using lyophilised mussels (0.2 g dm), spiked to achieve a final concentration of 125 ng g^−1^ dm for each target compound. The mussel samples were spiked by adding a methanol solution containing the selected antibiotics at the required spiking concentration in each case. The volume of the solution added was 200 μL, which was enough volume to impregnate the entire solid sample. Once the solution was added, it was vortexed for 1 min for homogenisation. The spiked mussel samples were incubated in the dark for 12 h to reach equilibrium and for the total evaporation of the methanol. All experiments were performed in triplicate.

#### 2.1.1. Optimisation of the Extraction Solvent

For the optimisation of the extraction solvent, an aprotic solvent (acetonitrile) and a protic solvent (methanol) were assessed. The use of water as extraction solvent required more than 12 h under a nitrogen stream for evaporation to dryness, which makes the method neither environmentally friendly, easy to perform, nor cheap without obtaining better recovery percentages of the analytes. For these reasons, the use of water was discarded.

**Figure 1:**
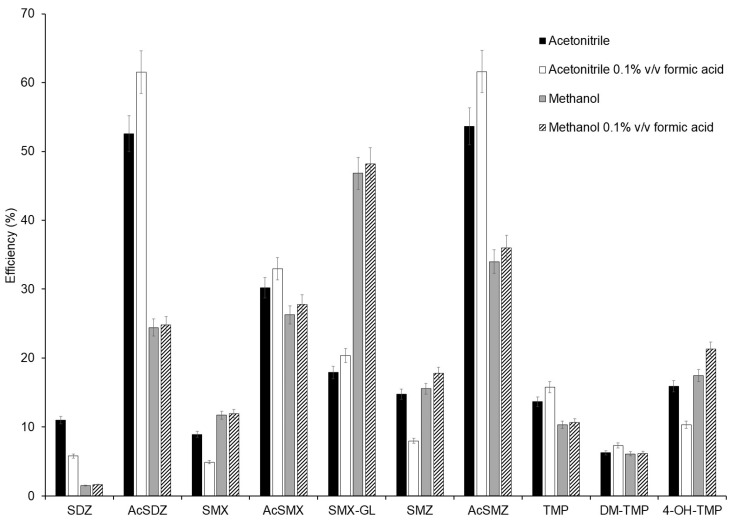
Extraction efficiency percentage of compounds obtained using different extraction solvents (*n* = 3).

These two solvents were tested as pure solvents and acidified at 0.1% *v*/*v* with formic acid. The initial conditions did not include sorbents for clean-up in the extraction procedure. For that, 0.2 g dm of spiked sample was mixed with 1 g of silica. The empty cartridge was fitted with a frit at the bottom. The mixture of the sample dispersed in the dispersant was placed on top of the frit and compacted in the cartridge, and another frit was added on top. The cartridge was eluted by gravity using three consecutive aliquots of 4 mL of each extraction solvent. Experiments were performed in triplicates. Mixtures of solvents were not optimised because previous works about the determination of sulphonamide antibiotics in similar samples revealed that better recoveries were obtained using a single solvent. For example, recently, Sun et al. (2023) [32] determined five sulphonamides in shrimp samples using acetonitrile as the extraction solvent. Similarly, Mu et al. (2022) [33] also employed only acetonitrile for the extraction of TMP and 17 sulphonamides from fish samples. Extraction efficiencies (peak area of spiked sample compared with the peak area of a standard at the same concentration in pure solvent) were calculated. Results can be seen in Figure 1. In most cases, acetonitrile showed better extraction efficiencies than methanol. It has been reported that acetonitrile has the ability to precipitate the proteins present in the matrix [33,34], obtaining better results in food samples. The addition of 0.1% *v*/*v* of formic acid to acetonitrile increased the extraction efficiencies but only in a few compounds, specifically in those that obtained better extraction efficiencies (such as N^4^-acetylsulfadiazine (AcSDZ) or N^4^-acetylsulfamethazine (AcSMZ)), while in the compounds that extracted worse the efficiency obtained was higher when acetonitrile alone was used. Given that the method proposed is a multiresidue approach, where compounds with different physical and chemical properties are analysed, it is necessary to achieve a balance that ensures optimal recovery for all analytes. For this reason, an extraction solvent was selected that, having good extraction for most of the compounds, best extracted the compounds with the worst extraction efficiencies. From these results, acetonitrile was selected as the extraction solvent for further experiments, as this solvent provided the best average results.

#### 2.1.2. Optimisation of d-SPE Sorbents and Their Amount

**Figure 2:**
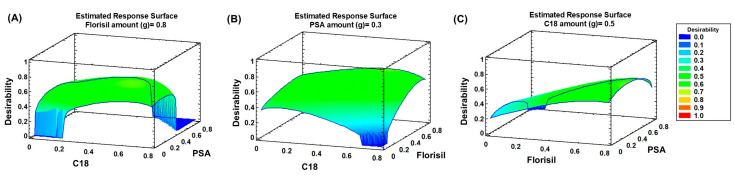
Response surface plots corresponding to desirability versus (**A**) C18 and PSA amounts (g); (**B**) C18 and Florisil amounts (g); (**C**) Florisil and PSA amounts (g).

Three sorbents were evaluated for extract clean-up. One reversed-phase sorbent (C18), one normal-phase sorbent (Florisil), and a weak anion exchanger sorbent (PSA). A Box–Behnken design (BBD) was applied to the clean-up optimisation, enabling a proper assessment of the influence and interactions of each variable. The number of experiments (N) necessary for BBD optimisation is calculated using Equation (1):

N = 2k(k − 1) + C0(1)

where k represents the number of variables, and C0 is the number of central points. Three variables were considered, corresponding to the types of clean-up sorbents assessed, with three central points. The experiments were conducted randomly. Each sorbent was tested at three levels (0, 0.4, and 0.8 g). Thus, 15 experiments were required for the simultaneous optimisation of the type and amount of sorbents. Table S1 presents the values assigned to each variable for each experiment. For that, 0.2 g dm of spiked sample was mixed with 1 g of silica. A mixture of the clean-up sorbents, according to the 15 experiments (Table S1), was placed on top of the frit. The sorbents were compacted in the cartridge, and another frit was added on top of them. The sample dispersed in the dispersant was then added on top of the second frit. It was compacted, and another frit was added on top of the sample. The cartridge was eluted by gravity using three consecutive aliquots of 2 mL of acetonitrile. Extraction efficiencies (peak area of spiked sample compared with the peak area of a standard at the same concentration in pure solvent) were calculated. BBD was calculated using the optimisation of multiple responses mode in Statgraphics 18-X64 version 18.1.16. Figure 2 illustrates the response surface plots of the geometric mean relative signals. As shown, a larger amount of Florisil (0.8 g) resulted in higher desirability. Regarding PSA, lower amounts yielded better desirability, with higher amounts producing worse results; the optimal amount was 0.3 g. Similarly, for C18, both very high and very low amounts resulted in lower desirability, with an intermediate amount (0.5 g) being optimal.

#### 2.1.3. Optimisation of Extraction Cycles

**Figure 3:**
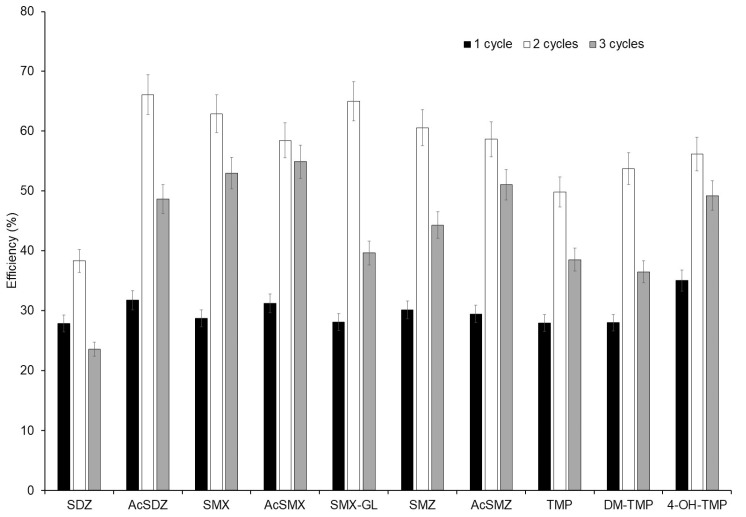
Extraction efficiency percentage of compounds obtained using different extraction cycles (*n* = 3).

In the optimisation of the technique, the remaining volume that could be added of extraction solvent, based on the capacity of the cartridge used and the amounts of sample, sorbent, and dispersants added, was 3.5 mL. The number of cycles (between 1 and 3) of extraction was therefore optimised, i.e., the number of times that 3.5 mL of elution solvent was added to the cartridge. For that, 0.2 g dm of spiked sample was mixed with 1 g of silica. An empty cartridge was fitted with a filter frit at the bottom. A mixture of the clean-up sorbents was placed on top of the frit, consisting of 0.8 g of Florisil, 0.5 g of C18, and 0.3 g of PSA. The sorbents were compacted in the cartridge, and another frit was added on top of them. The sample dispersed in the dispersant was then added on top of the second frit. It was compacted, and another frit was added on top of the sample. The cartridge was eluted by gravity using 1, 2, or 3 consecutive aliquots of 3.5 mL of acetonitrile. Experiments were performed in triplicates. Extraction efficiencies (peak area of spiked sample compared with the peak area of a standard at the same concentration in pure solvent) were calculated. Results can be seen in Figure 3. The highest extraction efficiency was obtained for two cycles. As expected, the higher the number of cycles, the higher the extraction efficiency when comparing one cycle and two cycles due to the higher volume of extraction solvent. The reduction in efficiency of the extraction from two cycles to three cycles can be attributable to the extraction of a major number of interferences with a high matrix effect that results in a low extraction efficiency. Therefore, two cycles were selected as the optimum.

### 2.2. Method Validation

The optimised methodology was validated for the determination of the antibiotics and their metabolites in mussel samples. The validation was conducted in terms of linearity, method detection limits (MDL), method quantification limits (MQL), precision (expressed as relative standard deviation, RSD), absolute recovery (R), and accuracy (A), expressed as relative recovery.

First, two different calibration curves were prepared: the first one in pure solvent (external calibration) and the other one by matrix-matched standards, both at six different concentration points and in triplicate. The slopes of both calibration curves were compared, and significant differences were obtained at 95% confidence using Student’s *t*-test. Therefore, the presence of matrix effects was confirmed, and a matrix-matched calibration curve was needed to be applied for the quantification of antibiotics. For this purpose, mussel samples were spiked in triplicate at six different concentration levels, and the final optimised methodology was applied to each of them. The ratio of the peak area after subtracting the blank peak area from the peak area of the internal standard was plotted versus the concentration of the analyte to obtain the matrix-matched calibration curve for the determination of antibiotics in mussel samples.

The determination coefficients (R^2^) for matrix-matched calibration curves with six different concentrations (from MQL to 125 ng g^−1^ dm) in triplicates were higher than 0.99 for all compounds (Table 1). MDL and MQL were obtained as the sample concentrations provided a signal-to-noise ratio of 3 and 10, respectively. For their calculation, spiked samples were employed. For all compounds, MQL values were in the range of 0.10 to 5.00 ng g^−1^ dm, and MDL values were from 0.03 to 1.50 ng g^−1^ dm. The obtained MDL, MQL, and matrix-matched calibration curve correlation coefficients (R^2^) values are presented in Table 1.

After that, matrix effects, recovery, precision, and accuracy values were calculated at three different concentration levels: 1.25, 12.5, and 125 ng g^−1^ dm, except for 4-hydroxytrimethoprim (4-OH-TMP), AcSDZ, and sulfamethoxazole N^4^-glucoside (SMX-GL), concentrations levels of which were 6.25, 12.5, and 125 ng g^−1^ dm.

The results obtained for recovery, accuracy, matrix effect, and precision at three spiking levels are shown in Table 2. The matrix effect was in the range from −56.4 to −2.14%. Matrix suppression was obtained in all compounds in mussel samples. Matrix suppression values were low considering the large amount of interferents that can be extracted from the mussel matrix, such as lipids or proteins. Recovery values ranged from 27.0 to 71.6% (Table 2). Accuracy ranged from 80.8 to 118% (Table 2). Analytical guidelines, such as those from the AOAC Peer-Verified Methods program [35], recommend recoveries ranging from 60 to 120% at part per billion levels, typically referring to absolute recovery or accuracy for individual analyses. Given that the method proposed in this work is a multiresidue approach, where compounds with highly varied properties are analysed, it is necessary to achieve a balance that ensures optimal recovery for all analytes. Furthermore, despite recoveries achieved for some of the target compounds being low (lower than 30%), as shown in Table 2, the accuracy reaches values between 80.8 and 120% for all analytes. This parameter and the low limits of quantification reflect the suitability of the method.

Precision was expressed as relative standard deviation (RSD, %). RSD values were below 17.7% for all compounds at the three spike concentrations (mean value: 10.0%) (Table 2).

Finally, the method’s selectivity was assessed by visualising potential interferences in the obtained chromatograms. No interference was observed at the retention times of the target compounds. Figure S1 shows the LC-MS/MS chromatogram of a 10 ng g^−1^ dm spiked mussel sample.

### 2.3. Method Comparison

Table 3 presents data on various analytical methodologies reported for determining antibiotics in bivalve mollusc samples.

Among the studied methods, mussels were the most frequently analysed molluscs, with clams and oysters also included. The proposed method used the smallest sample amount (0.2 g), while other methods required up to 2.0 g of sample. Various extraction techniques were employed (including MAE, PLE, UAE, and QuEChERS), but this work introduces the first method based on MSPD. Additionally, the proposed method used the lowest solvent volume (7 mL) compared with other methodologies, which used up to 200 mL [15]. For extract clean-up, Álvarez-Muñoz et al. (2015) [15] and McEneff et al. (2013) [16] employed an additional SPE step, while Fernández-Torres et al. (2010) [17] used LLE. The proposed methodology performed extraction and purification simultaneously, similar to the QuEChERS method reported by Serra-Compte et al. (2017) [9]. The main advancement of the developed MSPD method is the minimal amount of mussel sample and solvent required for the extraction of the target analytes and the simultaneous extraction and purification. Therefore, in terms of green sample preparation, the proposed method can be considered one of the most sustainable when compared with similar reported methodologies for analysing antibiotics residues in bivalve molluscs. Most methodologies used LC-MS/MS for antibiotic determination, whereas Fernández-Torres et al. (2010) [17] applied high-pressure liquid chromatography coupled with diode array detection and fluorescence detection (HPLC-DAD-FLD), which results in a loss of sensitivity, as it is the method with the highest MQLs. The cost of performing the technique is low as in the QuEChERS method in comparison with the other methodologies since it does not require the acquisition of high-cost equipment, as the PLE and MAE methods might require. Although at a lower cost, the method using UAE also requires expensive equipment. In addition, this technique does not require any additional steps such as sample centrifugation (which is necessary for MAE, UAE, and QuEChERS) since, thanks to the frits, the extraction solvent is filtered as it elutes from the cartridge. The extraction recoveries obtained with the proposed method were comparable to those of previously reported methods, even when using lower extraction solvent volumes. The dispersion of the matrix allows for a better extraction of compounds attributable to a better interaction between individual sample particles and the extraction solvent. The mechanical forces involved in mixing fragment the material into smaller parts with higher specific surfaces. Obtained MQL values were similar or lower in comparison with the previously reported methods, even when using smaller amounts of sample and extraction solvents.

### 2.4. Method Application

The proposed analytical method was applied to the monitorisation of sulphonamides and TMP in mussels exposed to target compounds. The results from the exposure assay are presented in Table S2. Field samples (mussels directly taken from the natural environment) showed concentrations below the MDL for all compounds. Consequently, samples at time 0 of the experiment (after a 7-day acclimation period but before antibiotic exposure) were also below the MDL for all compounds. After 14 days of exposure to 1 µg L^−1^ of SMX and TMP, the concentrations were 1.17 ng g^−1^ dm and 62.3 ng g^−1^ dm, respectively. These results indicate that TMP bioconcentrates more efficiently in mussels than SMX. This could be explained by the higher hydrophobicity of TMP, expressed by its higher octanol–water partitioning coefficient (log K_ow_ = 1.26), compared with SMX (0.79). An increase in SMX and TMP concentrations in mussels’ tissues was observed on the 28th day of exposure, reaching up to 1.26 ng g^−1^ dm and 77.5 ng g^−1^ dm, respectively, although no differences were observed compared with the 14th day. These findings underscore the significant health risks posed by the presence of antibiotics in the marine environment.

Serra-Compte et al. (2019) [36] revealed an increase in SMX concentration in mussels *M. galloprovincialis* up to 13.2 ± 0.7 ng g^−1^ dm after 96 h of exposure to the 10 µg L^−1^ of the antibiotic. The concentrations found in the whole soft tissues of mussels were 10 times higher, while the concentrations in seawater used were also 10 times higher, thus finding a 10-fold correlation. In contrast to the parent compounds detected in the whole tissues of mussels, no TMP- or SMX-related metabolites nor other sulphonamides and their metabolites were detected in the biological samples analysed herein. Similarly, none of the analytical approaches performed by Serra-Compte et al. (2019) [36] allowed the detection of SMX metabolites (i.e., SMX-GL and N^4^-acetylsulfamethoxazole (AcSMX)), suggesting that the parent compounds SMX and TMP were not metabolised by the selected marine species.

McEneff et al. (2014) [8] found residues of TMP at concentrations of 9.22 ng g^−1^ and 7.28 ng g^−1^ dm in the Mytilus spp. from organisms exposed to marine water naturally contaminated at 0.16 and 0.29 μg L^−1^, respectively. These concentrations were lower than those obtained in this study.

Bioconcentration is the process by which a pollutant is absorbed by an organism from the environment via the dermal and/or respiratory routes, with dietary intake not included. Bioconcentration factor (BCF, mL g^−1^) was calculated using measured concentration levels in seawater and biota using the following Equation (2):

BCF = C_b_/C_w_ (2)

where C_b_ is the concentration of a chemical in the biota (ng g^−1^ dm), and C_w_ is the concentration of a chemical in the water a (ng mL^−1^) at the same time at the end of the experiment (28th day). Calculated BCFs were 1.26 and 77.5 mL g^−1^ for SMX and TMP, respectively. Similar BCFs (up to 1.5 mL g^−1^) have been previously reported for SMX in mussels [36]. These results demonstrate that Mytilus galloprovincialis is a suitable bioindicator to assess marine pollution. In addition, it also highlights the high bioconcentration rates of antibiotics, especially TMP, in mussels, which can have negative effects on humans through the consumption of contaminated mussels. This scenario could worsen as the presence of antibiotics in mussels may promote the development of antimicrobial-resistant bacteria or genes and affect humans through the consumption of contaminated food products.

## 3. Materials and Methods

### 3.1. Chemicals and Reagents

Sigma-Aldrich (Steinheim, Germany) supplied high-purity standards of AcSDZ (≥99.0%), AcSMX (≥98.5%), sulfadiazine (SDZ, ≥99.0%), and sulfamethazine (SMZ, ≥99.0%). AcSMZ (≥98.0%), SMX-GL (>99.0%), 3-desmethyltrimethoprim (DM-TMP, ≥98.0%), and 4-OH-TMP (≥97.0%) were obtained from Toronto Research Chemicals (Toronto, ON, Canada). TMP (≥99.5%) and SMX (≥99.0%) were supplied by Dr. Ehrenstorfer GmbH (Augsburg, Germany). Physical–chemical properties of selected compounds can be seen in Table 4. The isotopically labelled compound, sulfamethoxazole-(phenyl-^13^C_6_) (SMX-^13^C, ≥99.0%), used as an internal standard, was supplied by Sigma-Aldrich (Steinheim, Germany). Florisil^®^, silica, and ammonium formate were provided by Sigma-Aldrich (Steinheim, Germany). Primary–secondary amine (PSA) and C18 were supplied by Scharlab (Barcelona, Spain). Formic acid (≥98.0%) was obtained from Panreac (Barcelona, Spain). All reagents were of high purity and analytical grade. Acetonitrile, methanol, and water of liquid chromatography–tandem mass spectrometry (LC-MS/MS) grade were supplied by Merck (Darmstadt, Germany).

### 3.2. Sample Collection and Exposure Assay

Mussels *M. galloprovincialis* (mean length: 6.82 ± 0.53 cm; mean width: 3.85 ± 0.30 cm) were harvested from a coastal farm in Sagres (Western Coast of Portugal), during winter (February 2024), and immediately transported alive to the laboratory. Upon arrival, specimens were cleaned by scraping off any fouling. The mussels were then placed in glass aquaria filled with 12.5 L of seawater (2 mussels per liter) with a salinity of 35 g L^−1^ and a temperature of 17 °C. They were kept under constant aeration and a light cycle of 12:12 h for a one-week acclimation period. The seawater was renewed every 48 h, and the mussels were fed with marine microalgae *Tetraselmis chuii*, cultured at a concentration of 150,000 cells per mussel, once per renewal day. After the acclimation period, the mussels were exposed for 28 days, in triplicate, to 1 μg L^−1^ of SMX and TMP. The light cycle was maintained at 12 h of light and 12 h of dark. The water was renewed every 48 h, with the antibiotic concentration re-established each time. During the exposure period, the mussels were also fed with *Tetraselmis chuii*, cultured at 150,000 cells per mussel, once per renewal day. Mussels were collected at 0, 14, and 28 days of exposure. They were sacrificed, and their tissues were freeze-dried in a Cryodos-50 lyophiliser (Telstar, Barcelona, Spain), homogenised in a mortar, and sieved (particle size < 100 μm). Lyophilised samples were frozen until analysis.

### 3.3. Sample Treatment

An MSPD technique was employed to extract the target analytes from the mussel samples. For that, 0.2 g dm of homogenised and lyophilised mussel sample was mixed with 1 g of silica (used as a dispersant/solid support). To achieve a thorough dispersion, the mixture was ground using a laboratory mini ball mill Pulverisette 23 (Fritsch, Idar-Oberstein, Germany) for 2 min at 35 oscillations per second. An empty SPE polypropylene cartridge of 6 mL was fitted with a polyethylene filter frit at the bottom. A mixture of the clean-up sorbents was placed on top of the frit, consisting of 0.8 g of Florisil, 0.5 g of C18, and 0.3 g of PSA. The sorbents were compacted in the cartridge, and another frit was added on top of them. The sample dispersed in the dispersant was then added on top of the second frit. It was compacted, and another frit was added on top of the sample. The cartridge was eluted by gravity using two consecutive aliquots of 3.5 mL of acetonitrile. To recover the maximum volume of extraction solvent, at the end of the elution process, a vacuum manifold system (Waters, Milford, AL, USA) connected to a vacuum pump was used. The eluate was collected and evaporated to dryness under a gentle nitrogen stream. The dried extract was then resuspended in 100 μL of methanol and 150 μL of water containing the internal standard and achieving a final concentration of 50 μg L^−1^, filtered with a syringe filter (0.22 μm) and collected in an automatic injector vial for LC–MS/MS determination.

### 3.4. Liquid Chromatography–Tandem Mass Spectrometry

Chromatographic determination was performed using an Agilent 1290 Infinity II chromatograph (Agilent, Palo Alto, CA, USA) equipped with a vacuum degasser, a binary pump, and an automatic injector. Chromatographic separation was conducted on a Zorbax RRHD Eclipse Plus C18 column (150 mm × 3.0 mm inner diameter, 1.8 μm particle size) (Agilent, Palo Alto, CA, USA), thermostated at 35 °C and protected with a Zorbax RRHD Eclipse Plus C18 guard column (3.0 mm inner diameter, 1.8 µm particle size) (Agilent, Palo Alto, CA, USA). The injection volume was 10 µL. The chromatographic conditions were those previously optimised [43]. The optimised LC-MS/MS conditions were as follows: gradient elution with a flow rate of 0.4 mL min^−1^ using a mobile phase composed of 10 mM ammonium formate (0.05% *v*/*v* formic acid) and methanol. Elution started with 5% methanol, maintained for 1 min, increased to 30% in 3 min, then to 60% in 8 min, and finally to 100% in 2 min, holding at 100% for 2 min. The return to initial conditions was achieved in 2 min and maintained for 2 min for re-equilibration. The LC system was coupled with a 6495 triple quadrupole (QQQ) mass spectrometer (Agilent, Palo Alto, CA, USA) equipped with an electrospray ionisation source. The mass spectrometry parameters were as follows: capillary voltage, 4000 V; fragmentor, 166 V; nebuliser pressure, 40 psi; sheath gas temperature, 250 °C; sheath gas flow rate, 12 L min^−1^; gas temperature, 350 °C; and gas flow rate, 11 L min^−1^. Analysis was performed in dynamic multiple reaction monitoring mode in positive ionisation mode. Therefore, the precursor ions corresponded to the molecular ions after protonation. The two most abundant transitions for each analyte were monitored, with the most abundant transition used for quantification and the other for confirmation. LC-MS/MS parameters for compounds can be seen in Table 5. LC–MS/MS parameters were optimised by injection of individual and mixture standard solutions of the selected compounds at 1 mg L^−1^. The type and composition of mobile phase solvents were optimised to achieve the highest compound ionisation to improve analytical signals and lower limits of detection.

Resolution of analytes was higher than 1.0 for all antibiotics, with the exception of the separation of SMZ and 4-OH-TMP. Even so, the differences in their precursor ion and product ions employed for their monitoring make it possible to perform the determination of all of them in just one injection.

### 3.5. Method Validation

The matrix effect was evaluated by comparison of the peak area of the target compounds in matrix extract (A_extract_), after subtracting the peak area obtained from non-spiked extracts (A_blank_), and in pure solvent standard solutions (A_standard_) applying Equation (3):

ME (%) = (A_extract_ − A_blank_ − A_standard_)/A_standard_ × 100) (3)

Extraction recoveries were assessed by comparison of the peak areas obtained from the spiked samples (A_sample_) with those from spiked extracts (A_extract_) after blank correction (A_blank_) following the Equation (4):

R (%) = (A_sample_ − A_blank_)/(A_extract_ − A_blank_) × 100 (4)

Accuracy (A), expressed as relative recovery, was determined by comparison of the concentration obtained from spiked samples using matrix-matched calibration curves (C_spiked sample_), after blank correction (C_blank_), with the spike concentration (C_spike concentration_) applying Equation (5):

A (%) = (C_spiked sample_ − C_blank_) × 100/C_spike concentration_ (5)

## 4. Conclusions

An MSPD-based method has been developed and validated for the first time for the determination of four antibiotics (TMP and three sulphonamides, namely SDZ, SMZ, and SMX) and six of their metabolites in mussel samples. Sample extraction and extract clean-up were performed in a single step and based on MSPD, using low volumes of solvent (7 mL) and sample amount (0.2 g). MQL values were in the range of 0.10–5.00 ng g^−1^ dm for all compounds. Accuracy, expressed as relative recovery, was in the range of 80.8–118%. Precision, expressed as relative standard deviation, obtained a mean value of 10%. The application of the method revealed that mussels can be suitable bioindicators of marine pollution since, after the exposure of the assay to TMP and SMX, concentrations in the mussels were found to be up to 77.5 ng g^−1^ dm. The bioconcentration of TMP in *M. galloprovincialis* was shown to be higher than that of SMX, attributable to its higher hydrophobicity.

The method was proven to be suitable for routine control of the presence of target antibiotics in mussel samples. Therefore, the proposed method offers a valuable tool for (i) obtaining information on the occurrence and fate of high-concern antibiotic classes and their metabolites in the marine environment, as mussels comprise suitable sentinel organisms applied for coastal pollution monitoring, (ii) supporting environmental risk assessments for antibiotic residues, (iii) evaluating the presence of antibiotics in edible tissues of mussels before human consumption, (iv) assessing their bioconcentration/bioaccumulation/biomagnification in mussels, and (v) revealing antibiotic overuse and environmental burden. Obtaining this information falls within the objectives of “One Health”, which is an approach to optimise the health of humans, animals, and ecosystems by integrating these fields rather than treating them separately.

## Figures and Tables

**Table 1:** Method detection limits (MDL), method quantification limits (MQL), and matrix-matched calibration curve correlation coefficients (R^2^).

Compound	Mussels		
	MDL (ng g^−1^ dm)	MQL (ng g^−1^ dm)	R^2^
**TMP**	0.30	1.00	0.997
4-OH-TMP	1.50	5.00	0.990
DM-TMP	0.30	1.00	0.997
**SMX**	0.03	0.10	0.998
AcSMX	0.30	1.00	0.994
SMX-GL	1.50	5.00	0.990
**SDZ**	0.15	0.50	0.996
AcSDZ	1.50	5.00	0.993
**SMZ**	0.03	0.10	0.999
AcSMZ	0.03	0.10	0.995

Parent compounds are marked in bold.

**Table 2:** Recovery (R%), accuracy (A%), matrix effect (ME%), and precision, expressed as relative standard deviation (RSD%), for mussels’ matrix at three spiking levels.

Compound	Mussels											
	1.25 (ng g^−1^, dm)				12.5 (ng g^−1^, dm)				125 (ng g^−1^, dm)			
	R (%)	A (%)	ME (%)	RSD (%)	R (%)	A (%)	ME (%)	RSD (%)	R (%)	A (%)	ME (%)	RSD (%)
**TMP**	28.1	102	−14.6	9.34	27.2	92.6	−26.8	17.7	33.1	118	−28.0	6.04
4-OH-TMP *	55.8	80.8	−12.2	11.6	45.0	98.4	−12.1	15.2	49.8	94.6	−20.1	5.14
DM-TMP	27.0	120	−54.2	7.41	27.5	97.5	−50.0	14.8	30.3	101	−44.6	12.3
**SMX**	57.5	98.0	−31.8	2.13	58.6	95.6	−26.0	2.52	54.7	96.6	−32.3	5.46
AcSMX	63.3	118	−20.7	13.8	63.3	89.4	−21.1	13.9	67.3	98.9	−3.42	7.20
SMX-GL *	53.2	101	−10.7	14.0	64.2	111	−56.4	17.4	53.7	93.5	−24.1	9.18
**SDZ**	28.5	109	−45.3	9.42	33.0	95.2	−45.3	3.80	29.4	100	−50.7	3.65
AcSDZ *	54.4	93.1	−2.14	13.2	54.3	102	−4.50	17.7	53.1	84.1	−6.51	17.3
**SMZ**	37.8	117	−12.3	6.53	36.3	88.0	−16.8	12.5	36.4	97.5	−17.6	6.32
AcSMZ	71.6	98.5	−4.12	14.9	71.4	86.4	−4.59	2.29	70.0	103	−14.8	7.80

Parent compounds are marked in bold; *: spiking levels: 6.25, 12.5, and 125 ng g^−1^ dm.

**Table 3:** Comparison of proposed methodology with other methods for determination of antibiotics in bivalve molluscs.

Compounds	Sample	Sample Amount (g)	Extraction	Solvent Volume (min)	Clean Up	Determination	Recovery	MQL (ng g^−1^, dm)	Reference
6 macrolides, 7 sulphonamides, metronidazole, TMP, 3 metabolites	Mussels and clams	0.5	QuEChERS	10	-	LC-MS/MS	28–60	0.05–1.03	[9]
3 nitroimidazoles, 1 sulphonamide, 2 macrolides, 1 metabolite	Mussels, oysters and clams	0.5	PLE	200	SPE	LC-MS/MS	30.2–115.7	0.02–2.66	[15]
2 b-lactams, 2 tetracyclines, 2 amphenicols, 5 sulphonamides, TMP and 5 metabolites	Mussels	2.0	UAE	15	LLE	HPLC-DAD-FLD	60.1–83.3	50–580	[17]
2 b-lactams, 2 tetracyclines, 2 amphenicols, 5 sulphonamides, TMP and 5 metabolites	Mussels	2.0	MAE	10	-	LC-MS/MS	63–97	5–55	[18]
TMP	Mussels	1.0	PLE	-	SPE	LC-MS/MS	91	4	[16]
3 sulphonamides, TMP and 6 metabolites	Mussels	0.2	MSPD	7	-	LC-MS/MS	27.0–71.6	0.1–5	Proposed methodology

-: no data; HPLC-DAD-FL: high-pressure liquid chromatography coupled to diode array detection and fluorescence; LC-MS/MS: liquid chromatography–tandem mass spectrometry; LLE: liquid-liquid extraction; MAE: microwave-assisted extraction; MQL: method quantification limits; MSPD: matrix solid-phase dispersion; PLE: pressurised-liquid extraction; QuEChERS: quick, easy, cheap, effective, rugged and safe method; SPE: solid-phase extraction; UAE: ultrasound-assisted extraction.

**Table 4:** Physical–chemical properties of the target compounds.

Compound	Molecular Weight (g mol^−1^)	p*K*_a_	Log *K*_ow_	Chemical Structure
**TMP**	290.3	7.16, 17.3 [37]	1.26 [37]	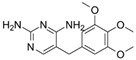
4-OH-TMP	306.3	8.18 [38]	-	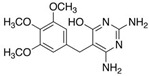
DM-TMP	276.3	9.40 [38]	-	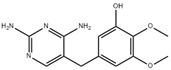
**SMX**	253.3	1.97, 6.16 [39]	0.79 [37]	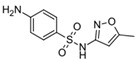
AcSMX	295.3	5.54 [40]	1.18 [40]	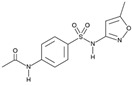
SMX-GL	415.4	-	-	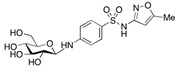
**SDZ**	250.3	2.01, 6.99 [37]	0.25 [37]	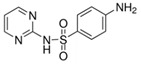
AcSDZ	292.3	6.10 [41]	0.39 [39]	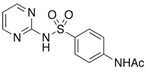
**SMZ**	278.3	2.04, 6.99 [37]	0.43 [37]	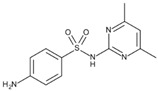
AcSMZ	320.4	7.16 [42]	1.48 [39]	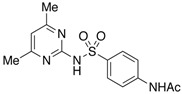

-: no data; parent compounds are marked in bold.

**Table 5:** LC-MS/MS conditions and retention times for the selected compounds.

Compound	Precursor Ion (*m*/*z*)	Product Ions (Quantifier/Qualifier) (*m*/*z*)	CE (eV)	Retention Time (min)	Ratio
**TMP**	291.2	261.1/229.8	28/24	7.79	98.2
4-OH-TMP	279.2	93.0/121.1	40/40	8.27	1.10
DM-TMP	277.3	261.4/123.0	28/44	6.80	63.1
**SMX**	254.3	92.1/108.0	28/28	8.96	76.1
AcSMX	296.3	134.0/108.1	24/28	10.74	49.8
SMX-GL	416.4	254.0/108.0	8/44	7.59	9.50
**SDZ**	251.3	92.1/156.0	28/12	6.45	98.0
AcSDZ	293.3	134.1/198.0	24/16	7.71	74.9
**SMZ**	279.3	186.0/92.0	16/36	8.28	76.4
AcSMZ	321.4	186.0/134.0	20/28	9.08	81.3
SMX-^13^C^IS^	260.2	98.1/162.0	32/16	8.95	94.5

CE: collision energy; IS: internal standard. Parents compounds are marked in bold.

## Data Availability

Data will be made available on request.

## Supplementary Materials

The following supporting information can be downloaded at https://www.mdpi.com/article/10.3390/molecules29225478/s1, Table S1: Box–Behnken design matrix for the optimisation of clean-up sorbent amount; Table S2: Method application to mussels (expressed as ng g^−1^ dm). Figure S1: LC-MS/MS chromatogram of a 10 ng g^−1^ dm spiked mussel sample.
