# Supplementary material for: Matrix Solid-Phase Dispersion Procedure for Determination of Antibiotics and Metabolites in Mussels: Application in Exposure Bioassays
Source: Molecules. 2024 Nov 20;29(22):5478. doi: 10.3390/molecules29225478 (PMC11597543; doi:10.3390/molecules29225478)
Supplement: Supplementary file 1 [file molecules-29-05478-s001.zip › molecules-3292882-supplementary.pdf]

Matrix Solid-Phase Dispersion Procedure for Determination of Antibiotics and Metabolites in Mussels: Application in Exposure Bioassays

Carmen Mejías [1], Tainá G. Fonseca [2], Noelia García-Criado [1], Julia Martín [1], Juan Luis Santos [1], Irene Aparicio [1] and Esteban Alonso [1,*]

[1] Departamento de Química Analítica, Escuela Politécnica Superior, Universidad de Sevilla, C/Virgen de África, 7, E–41011 Seville, Spain; cmpadilla@us.es (C.M.); ngarcia5@us.es (N.G.-C.); jbueno@us.es (J.M.); jlsantos@us.es (J.L.S.); iaparicio@us.es (I.A.)

[2] Centre for Marine and Environmental Research—CIMA/ARNET—Infrastructure Network in Aquatic Research, University of Algarve, Campus de Gambelas, 8000-139 Faro, Portugal; tgfonseca@ualg.pt

* Correspondence: ealonso@us.es; Tel.: +34-954-55-28-58

Table S1. Box–Behnken design matrix for the optimisation of clean-up sorbent amount.

Experiment	C18 amount (g)	PSA amount (g)	Florisil® amount (g)
1	0.8	0.4	0.8
2	0.8	0	0.4
3	0.4	0.4	0.4
4	0.4	0.8	0
5	0	0.8	0.4
6	0	0.4	0
7	0.8	0.8	0.4
8	0.4	0	0
9	0	0	0.4
10	0.4	0.4	0.4
11	0.4	0	0.8
12	0.4	0.8	0.8
13	0.4	0.4	0.4
14	0	0.4	0.8
15	0.8	0.4	0

Table S2. Method application to mussels (expressed as ng g^{-1} dm).

**Compound**	**Mussels**			
	$\mathbf{S_F}$	$\mathbf{S_0}$	$\mathbf{S_{14}}$	$\mathbf{S_{28}}$
**TMP**	-	-	62.3	77.5
4-OH-TMP	-	-	-	-
DM-TMP	-	-	-	-
**SMX**	-	-	1.17	1.26
AcSMX	-	-	-	-
SMX-GL	-	-	-	-
**SDZ**	-	-	-	-
AcSDZ	-	-	-	-
**SMZ**	-	-	-	-
AcSMZ	-	-	-	-

-: lower than MDL; Parent compounds are marked in bold; S_F: field sample; S_0: sample at time 0 (after acclimatisation period, before spiking); S_{14}: sample at time 14 days of exposure to SMX and TMP (1 μg L^{-1}); S_{28}: sample at time 28 days of exposure to SMX and TMP (1 μg L^{-1}).

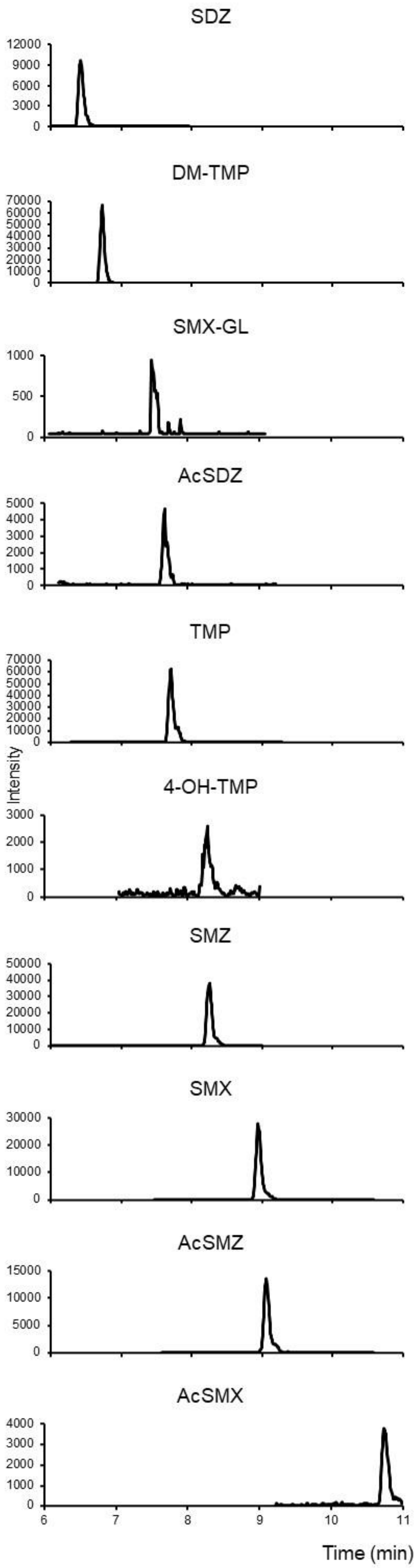

Figure S1. LC-MS/MS chromatogram of a 10 ng g^{-1} d.m. spiked mussel sample.
